# LUCID: Intelligent Informative Frame Selection in Otoscopy for Enhanced Diagnostic Utilitys

**DOI:** 10.21203/rs.3.rs-7502743/v1

**Published:** 2025-09-15

**Authors:** Hao Lu, Muhammet F. Demir, Gabriella I. Puchall, Zian Shang, Tucker Corwen, Shalaka Chavan, Carl D. Langefeld, Amy Zinnia, Muhammad Khalid Khan Niazi, Aaron C. Moberly, Metin N. Gurcan

**Affiliations:** a Center for Artificial Intelligence Research, Wake Forest University School of Medicine, Winston-Salem, NC, USA; b Dept. of Otolaryngology-Head and Neck Surgery, Vanderbilt University Medical Center, Nashville, TN, USA; c Department of Biostatistics and Data Science, Wake Forest University School of Medicine; d Department of Pathology, The Ohio State University

**Keywords:** Frame Selection, Middle Ear Diagnosis, Deep Learning, Eardrum Segmentation, Image Quality Assessment, Otologic AI

## Abstract

Accurate diagnosis of middle ear diseases, such as acute otitis media (AOM), remains a clinical challenge due to the reliance on subjective visual assessment through otoscopy. While deep learning has shown promise in improving diagnostic accuracy using digital otoscopy videos, existing models often rely on manually selected still frames, a step that reduces their practicality in real-world clinical workflows. In this study, we present the first systematic method (LUCID) for automatically identifying the most informative frame (MIF) selection in otoscopy videos. Through analyzing of 713 videos, we identified three key factors that impact frame informativeness: eardrum visibility, eardrum coverage, and image clarity. We then develop a novel MIF pipeline that integrates (1) a ResNet-50 classifier trained on over 38,000 labeled frames to assess eardrum visibility, (2) a binary-adversarial CAM (BC-AdvCAM) method for weakly supervised eardrum segmentation and coverage estimation, and (3) a specialized blur and focus detection algorithm tailored to otoscope imagery. These components are combined into an “informative score” to rank frames automatically. Comparative evaluations using human reviewers and diagnostic AI models show that frames selected by our AI method perform comparably to expert-selected frames—achieving similar classification accuracy across multiple deep learning architectures. Notably, using the top four frames per video identified by our method significantly improves diagnostic accuracy over using a single expert-selected frame. This framework offers a scalable, expert-level tool for automating key frame selection and enhancing AI-based otoscopy diagnosis.

## I. Introduction

Diseases of the ear, particularly acute otitis media (AOM) and middle ear effusions, are among the most common childhood pathologies, incurring an estimated annual financial burden of over $3.2 billion in the U.S. alone [1]. Accurate and timely diagnosis is crucial, as delayed or incorrect identification of ear disease can lead to significant complications, including the negative impact of hearing loss on children’s cognitive and language development [2]. In current diagnostic practice, clinicians rely on a visual assessment of the eardrum with an otoscope. However, this approach requires substantial clinical experience and remains prone to errors. Even experienced primary care, emergency medicine, and otolaryngology (ENT) physicians struggle to achieve high diagnostic accuracy, with reported accuracy rates ranging from a modest 50% to 80%, even when utilizing pneumatic otoscopy [3, 4]. This issue is particularly pronounced among clinicians with less otoscopy training, who account for the vast majority of clinical visits for ear complaints. For context, there are over 400,000 primary care clinicians in the U.S., compared to only 12,609 ENT physicians [5, 6].

To address the challenge of low diagnostic accuracy, many recent efforts have focused on developing machine learning (including deep learning) models to assist clinicians [7–18]. While promising, a significant limitation of most existing work is its reliance on single, well-selected still images for a machine learning model training and evaluation. In clinical practice, the clinician performs a dynamic ear exam over a few seconds or uses a digital otoscope to capture video sequences, not just static images. Consequently, prior machine-learning models typically necessitate a skilled clinician to manually select the most informative frame (MIF) from a video, a requirement that substantially limits their clinical applicability and scalability. Although some research explores video classification [19–21], these studies acknowledge that selecting MIFs can considerably improve performance. However, a systematic method for identifying and extracting the MIFs from otoscopy videos remains conspicuously absent in the literature.

This work presents, to the best of our knowledge, the first systematic and intelligent method to select the MIF from otoscopy videos. We engaged two ENT experts to manually select the MIF from a dataset of 303 otoscopy videos. During this meticulous selection process, we observed that less informative frames primarily suffered from three common issues: partial visibility of the eardrum, inadequate eardrum coverage, and image blur. Leveraging these insights, we propose a novel framework, **LUCID** (*Leveraging Useful Content in Informative Diagnostics*), which automatically selects the most diagnostically valuable frames by evaluating eardrum visibility, coverage, and image clarity.

To realize LUCID, we first trained a ResNet-50 model to detect the presence and view of the eardrum within an image. Second, we developed a binary-advCAM method, a weakly supervised model, to assess eardrum coverage using only image-level labels indicating the presence or absence of an eardrum. Third, we designed an otoscope-specific blur detection method based on edge energy analysis and focus evaluation. By combining these methodologies, we compute a comprehensive “informative score” for each frame. We then compare the frames automatically selected by our model with those manually chosen by ENT experts. Our findings indicate that while automatically selected frames may still be perceived as slightly inferior to human-selected frames in terms of subjective human assessment, they perform comparably when used as training data for deep learning models, yielding similar diagnostic accuracy.

Our key contributions are:

- The creation of a labeled dataset of 38,825 frames categorized as full eardrum, partial eardrum, or no eardrum, used to train a ResNet-50 model for eardrum view detection.
- The proposal of binary-advCAM, a weakly supervised model enabling eardrum segmentation with only image-level labels.
- The development of an otoscope-specific blur detection method, integrated with eardrum view and coverage assessments to compute a comprehensive “informative score.”
- A rigorous comparative analysis involving three expert clinicians and six different AI models to evaluate the quality of automatically selected frames versus human-selected frames.

The remainder of this paper is organized as follows: Section II reviews related work in image-based otoscopic diagnosis, video-based methods, and video quality assessment. Section III describes the methodology for eardrum view, coverage, and blur detection, and explains how these components are combined into an informative score. Section IV presents our experimental setup and quantitative results comparing expert-selected frames with those selected by the algorithm. Section V discusses the findings, limitations, and implications of our work. Finally, Section VI concludes the paper and outlines potential future directions.

## II. Related works

### II.1. Still Image-Based Diagnostic Assistance

Many studies have explored the use of high-quality, single-frame static images for diagnostic assistance in otoscopy [7–13]. These works primarily employ various deep learning architectures, including Xception, MobileNet, EfficientNet, and even YOLO, to automatically classify middle ear diseases from otoscope images. While the reported accuracies generally exceed 90% for specific diseases when trained and tested on well-curated datasets from the same source, a notable challenge arises when these models are evaluated on external datasets. For instance, studies by [8] and [10] demonstrated a significant drop in accuracy to approximately 75% when their models were tested on external datasets, highlighting concerns about generalizability. The use of these still-image-based models is highly dependent on a good selection of still frames from otoscope videos.

### II.2. Otoscope Video-Based Diagnosis

Recognizing that clinical digital otoscopy can involve video capture rather than just static images, some research has begun to utilize otoscope videos for diagnosis [19–21]. Approaches vary, with [20] focusing on generating two panoramic stitching images (composite images) from video frames to encompass the entire eardrum. This method achieved nearly 85% diagnostic accuracy on multi-center data. In contrast, [21] approached otoscopic diagnosis as a video classification task, employing a ResNet + LSTM architecture to differentiate between Acute Otitis Media (AOM) and normal cases, achieving an accuracy of 93%. Interestingly, both [20] and [21] mention the benefit of selecting MIF based on image clarity and contrast to improve classification performance. However, neither study provides a detailed investigation or systematic analysis specifically dedicated to MIF selection.

### II.3. Video Quality Assessment

The task of selecting informative frames from otoscope videos is closely related to the field of natural video quality assessment (VQA). VQA research broadly categorizes into subjective quality assessment, which involves human judgment of video quality [22–25], and objective assessment [26–31]. Subjective assessment is labor-intensive but provides crucial psychometric datasets that serve as benchmarks and labels for subsequent research. Objective assessment methods are further classified based on the availability of a reference video into Full-Reference (FR) VQA, Reduced-Reference (RR) VQA, and No-Reference (NR) VQA. Given that otoscope videos inherently lack a reference frame, our research falls under the No-Reference (NR) VQA paradigm.

Current NR VQA studies can be categorized into knowledge-driven models [32, 33] and deep learning-based methods [34–36]. Knowledge-driven approaches rely on handcrafted features to evaluate video quality. Deep learning-based methods, on the other hand, typically train deep learning models using subjective assessment results as labels.

However, our specific challenge extends beyond mere image quality; the selected frames must also contain diagnostic information relevant to the diagnosis. This requirement necessitates frame-level annotations from ENT experts, which are prohibitively expensive to obtain in sufficient quantities for training large deep learning models. Consequently, we adopt a hybrid approach combining knowledge-driven and deep learning methods. We train medical students with brief instruction to provide simple frame-level labels, indicating only whether a frame contains no eardrum, a partial eardrum, or a whole eardrum. These simplified labels are then used to train a classification model and an eardrum segmentation model. Finally, we employ handcrafted features to assess the clarity of the eardrum. To the best of our knowledge, this paper represents the first work to combine knowledge-driven and deep learning-based methods for addressing the challenge of key frame selection in otoscope videos, and it is also the first to analyze the quality of the selected key frames systematically. Building on these insights, we now describe the design and implementation of our proposed LUCID method.

## III. Method

This section details the proposed methodology for identifying diagnostically important frames from otoscope video sequences. The method systematically addresses three primary challenges that commonly render frames diagnostically unimportant: partial visibility of the eardrum, low eardrum coverage, and image blur. Our method combines deep learning for view and coverage assessment, along with a novel edge energy analysis for blur and focus evaluation, resulting in a comprehensive informative score.

### III.1. Dataset Acquisition and Annotation

The dataset utilized in this study comprises 713 high-definition otoscope videos, each representing a distinct ear examination. These videos were collected from a diverse array of clinical environments, encompassing adult and pediatric ear, nose, and throat (ENT) clinics, as well as operating rooms. Ethical approval for the study protocol was obtained from the local Institutional Review Board (IRB), with data collection conducted after written informed consent was obtained from patients and/or their guardians, ensuring adherence to all relevant ethical research guidelines (approval number IRB230109).

All video recordings were captured using the JEDMED Horus+ HD Video Otoscope, a high-definition digital otoscope known for its capability to acquire high-quality otoscopic imagery. Videos were stored in MPEG-4 format with a resolution of 1440 × 1080 pixels. Ground truth diagnoses for each case were rigorously established by a board-certified ENT specialist. This diagnostic process relied on microscopy in the clinic or operating room as the gold standard, further complemented by assessments of hearing status and eardrum mobility derived from audiometry and tympanometry.

Each video in the dataset corresponds to a unique ear, meaning a single patient could contribute either one or two videos, depending on whether one or both ears were examined. The final dataset is distributed across four primary diagnostic categories, as detailed in Table 1.

For evaluation purposes, two ENT experts manually selected the MIFs from a subset of randomly selected 303 videos. These expertly chosen MIFs serve as a crucial benchmark for comparison with the MIFs automatically selected by our proposed method. Furthermore, to facilitate training, three trained medical students annotated 38,825 frames from the remaining 410 videos (= 613 – 303). Each of these frames was meticulously labeled into one of three categories: “no eardrum,” “partial eardrum,” or “full eardrum.” These detailed annotations serve as the ground truth for training the eardrum classifier and eardrum segmentation model described in subsequent sections.

### III.2. Eardrum View Score: Addressing Partial Eardrum Visibility

As we mentioned, frames often present a partial or absent view of the eardrum, which contains less diagnostic information. To quantify the diagnostic importance related to eardrum visibility, we developed an “Eardrum View Score” based on a deep learning classification. We labeled 38,825 frames from 310 videos into three categories:

- **Full Eardrum:** The eardrum is completely visible within the frame.
- **Partial Eardrum:** Only a portion of the eardrum is visible.
- **No Eardrum:** The eardrum is entirely absent from the frame.

We use these data to train a ResNet-50 model.

Based on the classification probabilities (logits) output by the ResNet-50 model, a quantitative score for the eardrum’s presence and completeness is computed. We assign an “eardrum area score” to each class:

- Full Eardrum: Score of 1.0
- Partial Eardrum: Score of 0.5
- No Eardrum: Score of 0.0

The final Eardrum View Score ($S_{\mathrm{edv}}$) for a given frame is calculated as the expected value of the eardrum area scores, weighted by the classifier’s predicted probabilities for each class:

$$S_{\mathrm{edv}\;}=P_{\mathrm{full}}\times 1+P_{\mathrm{partial}\;}\times 0.5+P_{\mathrm{non}\;}\times 0$$

where $S_{\mathrm{edv}}$ denotes the eardrum view score, $P_{\mathrm{full}}$, $P_{\mathrm{partial}}$, and $P_{\mathrm{non}}$ represent the predicted probabilities of the frame containing a full, partial, or no eardrum, respectively. A higher $S_{edv}$ value indicates a more complete and diagnostically relevant view of the eardrum.

### III.3. Eardrum Coverage Score: Addressing Low Eardrum Coverage

Low coverage occurs when the eardrum occupies a small fraction of the total frame area, often due to a wide field of view, making detailed examination difficult. To address this, we need to segment the eardrum pixels. However, obtaining pixel-level eardrum labels requires significant expert effort, rendering ground truth annotation very expensive. Hence, we employ a weakly supervised semantic segmentation method that uses only image-level labels (indicating the presence or absence of an eardrum). We also used the data labeled as “no eardrum,” “partial eardrum,” and “full eardrum” from Section III.2. By grouping “partial eardrum” and “full eardrum” into a single “presence eardrum” category, we formulated a pixel-level binary classification problem.

#### III.3.1 Modified AdvCAM (BC-AdvCAM) for Weakly Supervised Semantic Segmentation

To perform Weakly Supervised Semantic Segmentation (WSSS), we adapted the AdvCAM [37] method. AdvCAM is designed to enhance Class Activation Maps (CAMs) for weakly supervised semantic segmentation. Traditional CAMs tend to focus only on the most discriminative regions of an object, which are sufficient for classification but fail to capture the full extent of the target object. AdvCAM addresses this limitation by employing adversarial climbing to expand object regions and regularization to suppress irrelevant activations, resulting in dense and accurate localization maps.

Adversarial climbing is the core of AdvCAM, which manipulates an input image iteratively to maximize the classification score for a target class. Unlike adversarial attacks that aim to deceive the model, adversarial climbing modifies the image to amplify the contribution of non-discriminative regions relevant to the target class.

The process begins with an input image $x_{0}$ and iteratively updates it using Eq. 1):

(1) $$x_{t}=x_{t-1}+\varepsilon \nabla_{x_{t-1}}L,$$

where:

(2) $$L=y_{c}-L_{\mathrm{class}-\mathrm{suppress}}-L_{\mathrm{restrict}},$$

(3) $$L_{\mathrm{class}-\mathrm{suppress}}=-\sum_{k\in C\backslash c}y_{k},$$

(4) $$L_{\mathrm{restrict}}=\lambda {\left\|M⊙CAM\left(x_{t}\right)-CAM\left(x_{0}\right)\right\|}_{1},$$

(5) $$M=1\left(CAM\left(x_{t-1}\right)>\tau \right),$$

And $x_{t}$ is the updated image at iteration t, $y_{c}^{t-1}$ is the classification score (logit) for the target class c at iteration t−1, $\nabla_{x_{t-1}}y_{c}^{t-1}$ is the gradient of the score with respect to $x_{t-1}$, ε is the step size controlling the magnitude of the update, C is the set of all classes, τ is a threshold, and 1(⋅) is an indicator function that identifies highly activated regions, ⊙ denotes element-wise multiplication, and λ is a weighting factor for the regularization. CAM refers to the class activation map of our model, computed using Grad-CAM.

This iterative process ensures that non-discriminative regions—those not initially involved in the classification—are gradually included in the model’s attention, thereby expanding the CAM to cover a larger portion of the target object.

The conventional AdvCAM formulation is typically used for multi-class problems; however, for our binary task, we modified its calculation to explicitly incorporate the background class, leading to more precise segmentation boundaries. The proposed binary class AdvCAM, BC-AdvCAM, formulation is as follows:

(6) $$x_{BC-ADvCAM}={AdvCAM}_{1}(x)-\lambda \times {AdvCAM}_{0}(x),$$

where

- $x_{BC-ADvCAM}$ represents the refined activation map for the eardrum class.
- $dv{CAM}_{1}(x)$ is the standard AdvCAM activation map for the eardrum (foreground) class.
- ${AdvCAM}_{0}(x)$ is the AdvCAM activation map for the background class.
- λ is a weighting factor that adjusts the influence of the background’s activation map. This parameter allows fine-tuning the suppression of background activations.

By subtracting a weighted background activation map, this formulation effectively suppresses irrelevant regions that might otherwise be activated in the foreground map, resulting in sharper eardrum boundaries. Notably, the influence of earwax, often present in the background, is captured in the ${AdvCAM}_{0}(x)$ map, and its suppression via subtraction enhances the focus on the eardrum’s true edges.

#### III.3.2 Coverage Score Calculation

After obtaining the segmented eardrum region, the Eardrum Coverage Score ($S_{\mathrm{edc}}$) is computed as the ratio of the eardrum pixels to the total number of pixels in the frame:

(7) $$S_{\mathrm{edc}}=\frac{\mathrm{eardrum}\;\mathrm{pixels}}{\mathrm{all}\;\mathrm{pixels}},$$

A higher $S_{\mathrm{edc}}$ value indicates that the eardrum occupies a larger and more diagnostically useful proportion of the image.

### III.4. Blur and Focus Score: Addressing Image Clarity

Blur detection in otoscope images is challenging, as traditional techniques (e.g., Fast Fourier Transform or Wavelet Transform) perform poorly. This reduced effectiveness stems from the inherent characteristics of otoscope images—specifically, their low variance and the concentration of image energy in low-frequency components. Our approach leverages edge energy distribution and a specific focus assessment to quantify image clarity.

#### III.4.1 Edge Energy Analysis for Blur Detection

**Figure 1.**
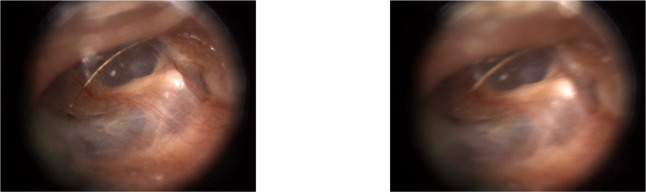

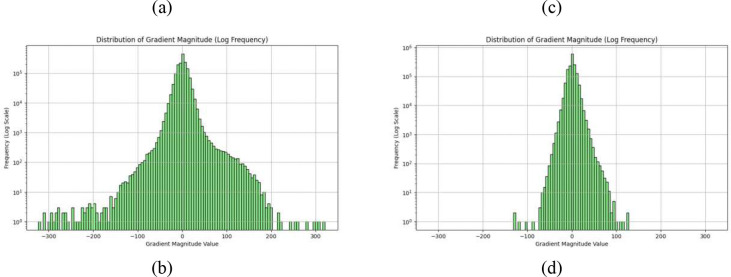
An example of the edge energy distribution for a sharp image ((a) and (b)) and a blurred image ((c) and (d)). The sign represents the edge direction.

We observed that clear otoscope images exhibit a “long tail” in their edge energy distribution, whereas blurry images show edge energy concentrated near zero, as illustrated in Fig. 1.

To leverage this distinction, we fit a Gaussian Mixture Model (GMM) with two Gaussian distributions to the computed edge energy distribution of a frame. A frame is deemed clear if one of the Gaussian distributions identified by the GMM possesses a large variance, indicating a broad spread of edge energies.

The initial blur score, denoted as $S_{bl}$, is computed as:

(8) $$S_{bl}=\frac{\sigma_{max}}{C_{p}\;+\;\epsilon },$$

where:

- $\sigma_{\max}$ is the standard deviation of the Gaussian distribution with larger variance, indicating the spread of edge energies.
- $C_{p}$ is contrast of given image.
- ϵ is a small constant added to the denominator (e.g., 10^−6^) to prevent division by zero.

A small $S_{bl}$ indicates a blurry image, characterized by a large $C_{p}$ (due to local contrast that might still exist in some areas) but a small $\sigma_{max}$ (meaning concentrated edge energy). Conversely, a larger $S_{bl}$ implies a clearer image.

#### III.4.2 Otoscope Field of View and Focus Point Detection

Often, the otoscope may not be perfectly focused on the eardrum, but rather on other structures within its circular field of view (FOV). To ensure the focus is diagnostically relevant (i.e., near the center of the eardrum), we first identify the otoscope’s circular FOV and then determine the focus point relative to its center.

For the FOV Detection, we use the Hough circle transform to identify the circular FOV. The circle with the most extensive lightness changes between its interior and exterior is selected as the most probable representation of the otoscope’s FOV.

In detail, let I(x,y) be the intensity (or lightness) value of the image at pixel coordinates (x,y). The Hough Circle Transform is applied to identify potential circular structures in the image. Let the set of detected circles be $C=C_{k}$, where each circle $C_{k}$ is defined by its center coordinates ($x_{ck},y_{ck}$) and its radius $r_{k}$.

For each detected circle $C_{k}$, we calculate the average lightness within its interior and within a defined exterior region (typically an annulus immediately surrounding the circle). The interior region ($R_{\mathrm{int},k}$) is the set of pixels (x,y) that lie within or on the boundary of the circle $C_{k}$.


(9) $$R_{int,k}=\left\{(x,y)|{\left(x-x_{ck}\right)}^{2}+{\left(y-y_{ck}\right)}^{2}\leq r_{k}^{2}\right\},$$

And the average interior lightness ($L_{\mathrm{int},k}$) is define as the mean intensity of pixels within the interior region.

(10) $$L_{int,k}=\frac{1}{\left|R_{int,k}\right|}\sum_{\left(x,y\right)\in R_{int,k}}I\left(x,y\right),$$

where $\left|R_{\mathrm{int},k}\right|$ is the number of pixels in the interior region.

The exterior annulus region ($R_{ext,k}$) is defined as the set of pixels (x,y) that lie within a small annulus immediately outside the circle $C_{k}$. Let Δr be a small, predefined width for this annulus.


(11) $$R_{ext,k}=\left\{(x,y)|r_{k}^{2}<{\left(x-x_{ck}\right)}^{2}+{\left(y-y_{ck}\right)}^{2}\leq {\left(r_{k}+\Delta r\right)}^{2}\right\},$$

Same as the average interior lightness, the average exterior lightness ($L_{\mathrm{ext},k}$) is defined as

(12) $$L_{ent,k}=\frac{1}{\left|R_{ext,k}\right|}\sum_{\left(x,y\right)\in R_{ext,k}}I\left(x,y\right),$$

And the lightness change measure ($\Delta L_{k}$) is defined as the absolute difference between the average interior and exterior lightness values, $\Delta L_{k}=\left|L_{\mathrm{int},k}-L_{\mathrm{ext},k}\right|$.

The circle with the highest calculated lightness change is selected as the most probable representation of the otoscope’s Field of View ($C_{FOV}$).


(13) $$C_{FOV}=\arg\underset{C_{k}\in C}{max}\left(\Delta L_{k}\right),$$

For the focus point detection, we used the Laplace transform, combined with a Gaussian filter, to identify the focus point within the image. In detail, the image is first smoothed by convolving it with a 2D Gaussian kernel to reduce noise and provide multi-scale analysis. 2D Gaussian Kernel (G) is defined as $G(x,y,\sigma )=\frac{1}{2\pi \sigma^{2}}e^{-\frac{x^{2}+y^{2}}{2\sigma^{2}}}$, where σ is the standard deviation, controlling the extent of smoothing. Hence, the smoothed image 𝐼 with the Gaussian kernel is

(14) $$I_{G}\left(x,y\right)=I\left(x,y\right)\;*\;G\left(x,y,\sigma \right),$$

where * denotes the convolution operation.

The Laplacian operator ($\nabla^{2}$) is then applied to the Gaussian-smoothed image to detect regions of rapid intensity change (edges or sharpness). For a 2D function, the Laplacian is defined as the sum of its second partial derivatives.


(15) $$\nabla^{2}I_{G}(x,y)=\frac{\partial^{2}IG(x,\;y)}{\partial x^{2}}+\frac{\partial^{2}IG(x,\;y)}{\partial y^{2}},$$

Regions of high absolute response in the ILoG(x,y) image correspond to sharp intensity transitions, indicating high spatial frequency content often associated with focus.

A common method is to use the absolute value or the squared value of the LoG response as a sharpness measure at each pixel.


(16) $$\left(x_{f},y_{f}\right)=\arg\underset{\left(x,y\right)}{max}\;S\left(x,y\right),$$

Where $S(x,y)={\left(\nabla^{2}I_{G}(x,y)\right)}^{2}$.

The Focus Score ($S_{F}$) quantifies how centrally the image is focused within the detected otoscope FOV. It is computed as:

(17) $$S_{F}=r_{FOV}-\sqrt{{\left(x_{f}-x_{FOV}\right)}^{2}+{\left(y_{f}-y_{FOV}\right)}^{2},}$$

A higher Focus Score indicates that the sharpest region of the image is closer to the center of the otoscope’s field of view, suggesting diagnostically relevant focus.

#### III.4.3 Final Blur Score

The final Blur Score, $S_{blf}$, for a frame is a composite of the initial blur assessment and the focus assessment:

(18) $$S_{blf}=S_{bl}\times S_{F},$$

A higher $S_{blf}$ value signifies a clearer image with a diagnostically relevant focus.

### III.5. Informative Score Calculation

The final informative score, $S_{I}$, integrates the assessments from eardrum view, eardrum coverage, and blur and focus into a single metric. This score provides a comprehensive measure of a frame’s diagnostic utility. We use weighted sum of the ranks of the individual scores to compute $S_{I}$:

(19) $$S_{I}=0.4\times Rank\left(S_{edv}\right)+0.2\times Rank\left(S_{edc}\right)+0.4\times Rank\left(S_{blf}\right),$$

Here, Rank(·) refers to the ranking of a particular score among all frames, indicating its relative position in terms of view quality. The weighting factors (0.4 for Eardrum View Score, 0.4 for Final Blur Score, and 0.2 for Eardrum Coverage Score) were determined experimentally to balance their respective contributions to overall diagnostic importance.

A smaller informative score $S_{I}$ indicates a more diagnostically informative frame. This inverse relationship ensures that frames with optimal eardrum visibility, comprehensive coverage, and high clarity are prioritized.

## IV. Experiments and Results

### IV.1. Experiments setup

The ResNet50 trained for Eardrum View Score was trained with 38,825 frames from 310 videos. This dataset was preprocessed by resizing images to 224×224 pixels, converting them to tensors, and normalizing them using ImageNet means and standard deviations. The 310 videos were then split into training (70%), validation (15%), and test (15%) sets. Data loaders were configured with a batch size of 32. The model employed was a pre-trained ResNet50, with its final classification layer adapted to the 3 (“no eardrum”, “partial eardrum”, or “full eardrum”) in our dataset. The model was trained for 25 epochs using Adam optimizer with a learning rate of 0.001 and Cross-Entropy Loss. Model selection was based on the highest accuracy achieved on the validation set. The model trained for Modified AdvCAM was trained in the same way. The only difference is the final layer adapted to 2 (“no eardrum” and “with eardrum”). Both foreground and background AdvCAM were both iterated 30 times.

### IV.2. Eardrum visibility classification result

Table 2 presents the confusion matrix for the three-class classification task (“no eardrum,” “partial eardrum,” and “full eardrum”) on the test dataset.

### IV.3. Eardrum segmentation result

As we discussed in Section III.2, to get the coverage score $S_{EDC}$, we need to segment the eardrum using our proposed BC-AdvCAM method. The segmentation results are presented in Tables 3 and 4, which compare our proposed BC-AdvCAM method with baseline models, including Grad-CAM, AdvCAM, and SAM with different numbers of positive point prompts (SAM_p_0 to SAM_p_3), where the positive point prompts are randomly selected within the eardrum area based on the ground truth segmentation. The performance is assessed using multiple evaluation metrics to provide a comprehensive comparison of segmentation effectiveness. The evaluation metrics include, IoU: Intersection over Union, measuring overlap between predicted and ground truth masks; DSC: Dice Similarity Coefficient, a common metric for evaluating segmentation quality; Acc: Accuracy, the proportion of correctly predicted pixels; Sen: Sensitivity, the ability to detect positive regions; Spe: Specificity, the ability to avoid false positives; AUROC: Area Under the Receiver Operating Characteristic curve, the ability to distinguish between positive and false region; HD: Hausdorff Distance, measuring the distance between predicted and ground truth boundaries; MAD: Mean Absolute Deviation, quantifying the average pixel-level difference.

To test whether these estimates are statistically different while accounting for the potential correlation among images from the same person, we computed generalized estimating equations (GEE1) using the sandwich estimator of the variance. Even after adjusting for multiple comparisons, these results show strong differences among the performance metrics, except sensitivity for Grad-CAM.

### IV.4. Most informative frame selection result

As previously mentioned, two ENT experts manually selected the MIF from a subset of 303 otoscopy videos. We then utilized the method described in Section 3 to extract MIFs from the same set of videos automatically. To compare the expert-selected MIFs with those selected algorithmically by our approach, we employed two distinct evaluation methods.

**Figure 2.**
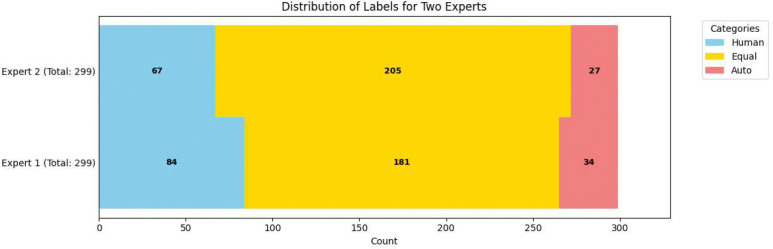
Human Assessment of MIF Selection. This stacked bar chart illustrates the independent human evaluation of automatically selected (Auto) versus expert-selected (Human) Most Informative Frames (MIFs) from 299 otoscopy videos. Blue indicates reviewer preference for the expert-selected MIF, red for the algorithm-selected MIF, and yellow for cases where both were considered equivalent or indistinguishable.

**Figure 3.**
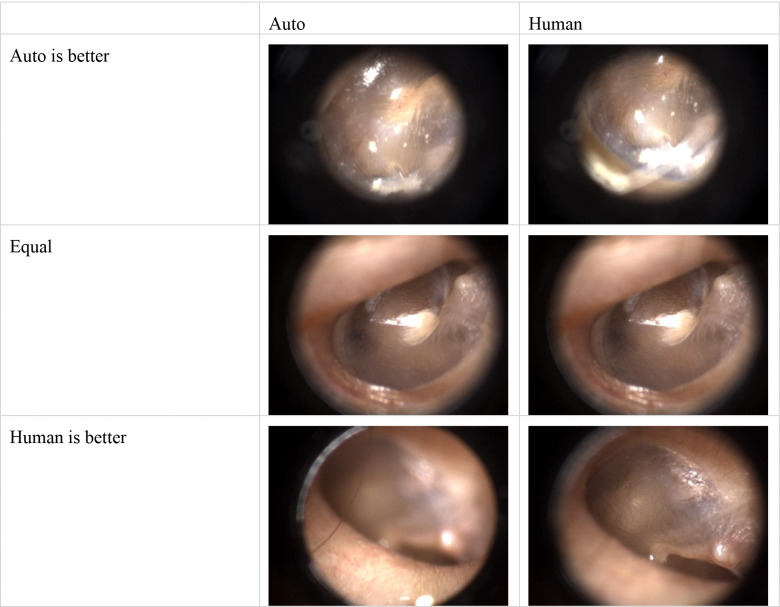
Examples of both reviewer believes Auto-selected MIF is better; Equal good; and human-selected MIF is better.

An emergency physician and a trained medical student, both with otoscopy training, independently evaluated 299 pairs of MIFs, comprising one expert-selected and one algorithm-selected frame each (four videos were excluded due to transmission errors). The results are depicted in Figure 2. Blue segments indicate instances where the human reviewer preferred the expert-selected (human) MIF, while red segments show preference for the algorithm-selected (auto) MIF. Yellow segments represent cases where the reviewers deemed both MIFs equivalent or indistinguishable. On average, reviewers considered the algorithm-selected MIF to be at least equivalent to the expert-selected MIF in approximately 74% of the videos. Figure 3 shows the comparison between Auto selected image and Human selected images.

Second, we trained four common image classification models (ResNet34, ConvNeXt tiny, Swin Transformer tiny, and ViT B/16) for the automated diagnosis of otoscopy images. This constituted a four-class classification task: Normal, Effusion, Perforation, and Tympanosclerosis. For each model, we used MIFs from 227 videos for training, 30 videos for validation, and 46 images for testing. Importantly, each model was trained separately using either the expert-selected (human) MIFs or the algorithm-selected (auto) MIFs as the training dataset. We employed Bayesian optimization, iterating 40 times on the validation set for each model to fine-tune hyperparameters and ensure optimal performance.

**Figure 4.**
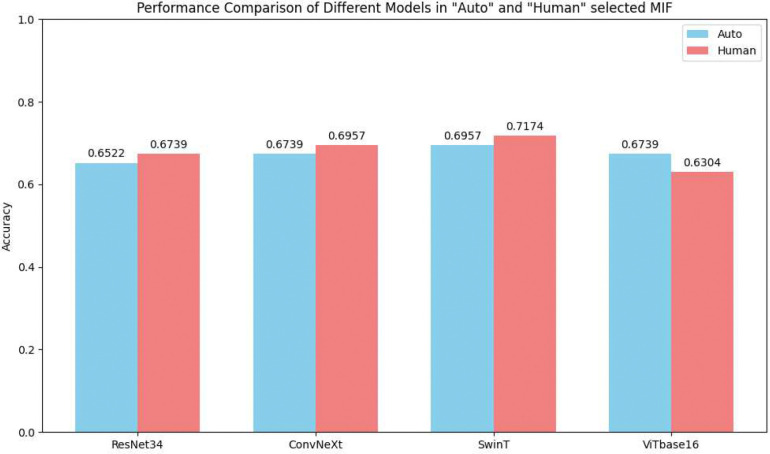
Comparative Performance of Automated Diagnostic Models Trained on Different MIF Sources. Red bar for expert-selected (Human) dataset and Blue bar for automatically selected (Auto) dataset.

Figure 4 illustrates the comparative performance of these automated diagnostic models when trained on expert-selected versus algorithm-selected MIFs. The results indicate that models trained on automatically selected MIFs performed comparably to those trained on expert-selected MIFs. Specifically, for ResNet34, ConvNeXt tiny, and Swin Transformer tiny, training with algorithm-selected MIFs resulted in only a 2% reduction in accuracy compared to using expert-selected MIFs. Notably, the ViT B/16 model even exhibited better convergence when trained on the automatically selected MIFs.

### IV.5. Human Preference Analysis

To further evaluate how human reviewers perceive the relative quality of algorithm-selected versus expert-selected frames, we conducted three statistical tests using the human preference annotations from 299 video pairs (each pair containing one expert-selected frame and one algorithm-selected frame):

#### Test 1: Preference Distribution Test

We conducted a chi-square goodness-of-fit test (df = 2) to determine whether the three preference options—“Auto,” “Human,” and “Equal”—occurred with equal frequency. Under the null hypothesis, each preference would be expected to occur in approximately equal proportion (i.e., 299/3 ≈ 99.67 per category). The resulting p-values for both reviewers were less than 1 × 10^−25^, strongly rejecting the null hypothesis and indicating that the preferences are not uniformly distributed.

#### Test 2: Agreement vs. Disagreement

We grouped “Equal” responses (both frames considered similar) as “Agree” and the remaining (“Auto” or “Human”) as “Disagree.” A one-sample binomial test was performed to evaluate whether the frequency of “Agree” was significantly different from chance (p = 0.5). Both reviewers yielded p-values < 1 × 10^−4^, suggesting that a substantial number of videos were labeled as “Equal,” indicating that many algorithm-selected frames were perceived as equally informative as auto-selected and human-selected ones.

#### Test 3: Auto vs. Human Preference (Conditional on Disagreement)

Conditioned on disagreement (selecting either “Auto” or “Human”), we examined whether reviewers were more likely to prefer “Human” over “Auto.” Using a one-sample binomial test (with success defined as preferring “Human”), both reviewers yielded p-values < 1 × 10^−5^, indicating that when a distinction was made, human-selected frames were more often preferred.

These results reveal two important findings: (1) a large portion of algorithm-selected frames are indistinguishable in quality from expert-selected frames, and (2) when differences are observed, expert-selected frames still hold a slight advantage in perceived informativeness. However, this subjective preference does not significantly impact model training performance, as shown in our classification experiments.

## V. Discussion

The primary aim of this study was to develop and evaluate a systematic, intelligent informative approach for selecting the MIFs from otoscopy video sequences. This addresses a significant practical limitation of current deep learning models for otoscopy diagnosis, which often rely on manual, expert-driven frame selection. Our findings provide strong evidence for the effectiveness and potential clinical utility of our proposed framework.

While our algorithm’s frame selection capabilities may still exhibit a gap when compared to expert human judgment, it’s crucial to consider the substantial cost associated with expert manual annotation. More importantly, our method demonstrates comparable performance to expert-selected frames when used to train diagnostic models. Furthermore, our approach can score and rank all frames within a video. For instance, training a ResNet34 model using the top four MIF per video, as identified by our algorithm, increased diagnostic accuracy to 77.57%. This represents a considerable improvement over models trained solely on single, manually selected frames, which achieved an accuracy of 67.39%. This highlights the potential of our method to leverage multiple high-quality frames from a video, thereby enhancing diagnostic performance beyond what single-frame selection can achieve. It should be noted that these accuracy rates are lower than those reported in our previous work because only 303 images were available in this study for training (limited to the ground truth frames collected). The comparison here is intended primarily to demonstrate relative improvement rather than absolute performance.

**Figure 5.**
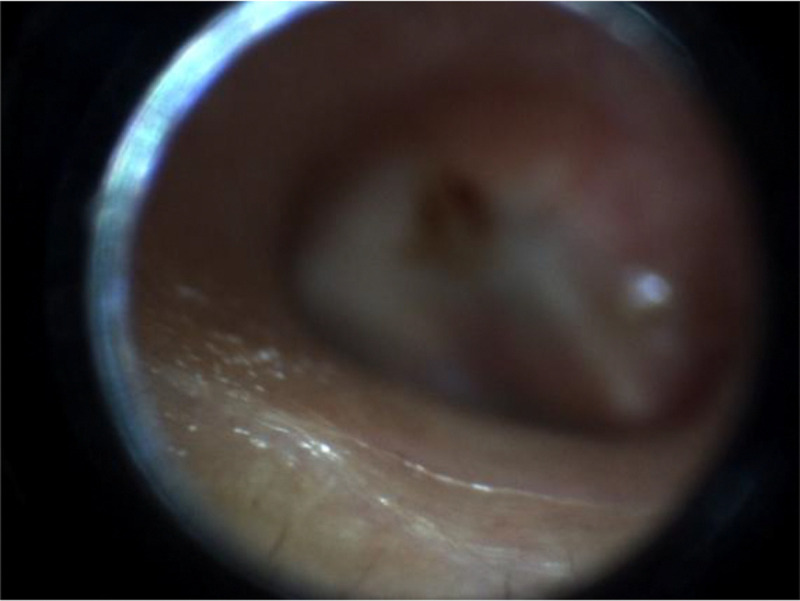
Example of Algorithm Failure in MIF Selection.

Despite generally stable performance in identifying informative frames, our method has limitations. The accuracy of our eardrum segmentation and eardrum clarity analysis is inherently constrained by the weakly supervised and unsupervised learning approaches employed. For example, as illustrated in Figure 5, our algorithm may occasionally misinterpret frames where the focus is on a region other than the eardrum. In such instances, the limited eardrum segmentation capability might still identify the focused area as the eardrum, and the algorithm would incorrectly rate the image as clear and well-focused within the field of view, potentially resulting in an incorrectly identified MIF. In addition, our dataset includes a mix of pediatric and adult otoscopy videos, which introduces variability in anatomy, pathology presentation, and image quality. While our method performed reasonably well across both groups, the heterogeneous age distribution may affect generalizability, and future work should investigate whether separate or age-specific models could further improve accuracy.

Looking ahead, we envision this research serving as a foundational auxiliary tool to enhance the capabilities of automated otoscopy diagnosis. Simultaneously, it can function as an assistance tool for annotating otoscopy MIFs, enabling experts to label and build larger, more comprehensive datasets rapidly. Such datasets, in turn, can facilitate the training of even more precise and robust MIF selection models in the future.

## VI. Conclusion

This study successfully developed and evaluated a novel, systematic framework for intelligent MIF selection from otoscopy videos, addressing a key limitation in AI-assisted diagnosis. Our method, which assesses eardrum visibility, coverage, and image clarity, yielded MIFs comparable to expert selections, both in human judgment and, critically, in their effectiveness for training diagnostic AI models.

Notably, human-selected frames were still slightly preferred in subjective review, reflecting the nuanced expertise of clinicians. However, this preference did not affect model training performance: diagnostic models trained on algorithm-selected frames achieved accuracy comparable to those trained on expert-selected frames. Furthermore, leveraging our method’s ability to rank frames, using the top four MIFs significantly boosted diagnostic accuracy (e.g., 77.57% with ResNet34 **vs.** 67.39% with single manual frames). This demonstrates the framework’s practical and scalable value in both AI development and expert annotation.

However, this study has several limitations. First, the segmentation of eardrum regions was achieved using weakly supervised learning due to the lack of pixel-level annotations, which can limit the precision of coverage estimation. Second, our blur and focus detection algorithm, while tailored to otoscopic images, may still fail in complex scenarios such as specular reflection, occlusion by wax, or off-center focus. Third, although we tested our method on a substantial dataset, further validation on larger and more diverse populations, including different device types and clinical settings, is warranted to confirm generalizability.

For future work, we plan to integrate temporal information across video frames to enhance the robustness of frame selection. Additionally, we aim to incorporate clinical metadata to guide the learning process further. Expanding expert annotation efforts will also enable us to train fully supervised segmentation models and improve blur detection using more advanced self-supervised or foundation models. Ultimately, we envision incorporating this framework into real-time otoscope workflows to provide on-the-fly frame selection and diagnostic support.

## Figures and Tables

**Table 1.** Distribution of otoscopy videos by diagnostic category

Category	Number of videos

Normal	323
Effusion	256
Perforation	68
Tympanosclerosis	66

**Total**	**713**

**Table 2.** Confusion Matrix for Eardrum Classification on the Test Set

_True Label_╲^Predicted Label^	no eardrum	partial eardrum	full eardrum
no eardrum	99.41%	0.24%	0.35%
partial eardrum	0.45%	99.40%	0.15%
full eardrum	0.09%	0%	99.91%

**Table 3.** Comparison of segmentation performance for different methods, including Grad-CAM, advcam, our proposed BC-AdvCAM, and SAM with varying positive point prompts (SAM_p_0 to SAM_p_3). ↑ indicates that larger values are better, and ↓ indicates that smaller values are better.

Method	IoU ↑	DSC ↑	Acc ↑	Sen ↑	Spe ↑	AUROC ↑	HD ↓	MAD ↓
Grad-CAM	0.5436	0.6753	0.8805	0.7834	0.8969	0.8402	38.4291	1.8078
Adv-CAM	0.6176	0.7297	0.8858	0.8795	0.8760	0.8778	35.2936	0.3229
BC-AdvCAM	**0.6717**	**0.7924**	**0.9091**	0.8546	**0.9282**	**0.8914**	**32.2179**	1.1937
SAM_p_0	0.0911	0.1364	0.4258	0.3101	0.4553	0.3827	124.2249	38.1030
SAM_p_1	0.3704	0.5193	0.5799	0.9987	0.4591	0.7289	91.2550	0.0029
SAM_p_2	0.4088	0.5603	0.6521	0.9989	0.5511	0.7750	83.8071	0.0058
SAM_p_3	0.4338	0.5859	0.6911	**0.9996**	0.5999	0.7998	78.7163	**0.0008**

**Table 4.** Generalized Estimating Equation (GEE) analysis, considering correlation within patients using AUC and various metrics, with an exchangeable correlation structure and identity link.

measurement	Delta = BC_AdvCAM - X	Estimate	Estimate SE	P-value
IoU	Adv-CAM	0.0466	0.0164	0.0045
IoU	Grad-CAM	0.0882	0.0158	2.47 × 10^−8^
DSC	Adv-CAM	0.0384	0.012	0.00139
DSC	Grad-CAM	0.0671	0.0122	3.93 × 10^−8^
Accuracy	Adv-CAM	0.0341	0.0065	1.59 × 10^−7^
Accuracy	Grad-CAM	0.0352	0.0056	3.16 × 10^−10^
Sensitivity	Adv-CAM	−0.1227	0.0109	<2 × 10^−16^
Sensitivity	Grad-CAM	0.0102	0.0178	0.56
Specificity	Adv-CAM	0.0797	0.0064	<2 × 10^−16^
Specificity	Grad-CAM	0.0371	0.0059	4.10 × 10^−10^
AUC	Adv-CAM	−0.0215	0.0065	9.80 × 10^−4^
AUC	Grad-CAM	0.0237	0.0085	0.00552
HD	Adv-CAM	−7.4465	1.9005	8.92 × 10^−5^
HD	Grad-CAM	−6.4042	1.2581	3.58 × 10^−7^
MAD	Adv-CAM	1.0975	0.1699	1.06 × 10^−10^
MAD	Grad-CAM	−0.5793	0.2849	0.0420

## References

[1] Soni A., “The five most costly children’s conditions, 2011: Estimates for US civilian noninstitutionalized children, ages 0–17,” in “ Statistical Brief (Medical Expenditure Panel Survey (US))[Internet].”, 2018.

[2] Physicians A. A. o. F., Otolaryngology-Head A. A. o., Surgery N., and Effusion A. A. o. P. S. o. O. M. W., “Otitis media with effusion,” Pediatrics, vol. 113, no. 5, pp. 1412–1429, 2004. 15121966 10.1542/peds.113.5.1412

[3] Rogers D. J., Boseley M. E., Adams M. T., Makowski R. L., and Hohman M. H., “Prospective comparison of handheld pneumatic otoscopy, binocular microscopy, and tympanometry in identifying middle ear effusions in children,” International journal of pediatric otorhinolaryngology, vol. 74, no. 10, pp. 1140–1143, 2010. 20638734 10.1016/j.ijporl.2010.06.015

[4] Sorrento A. and Pichichero M. E., “Assessing diagnostic accuracy and tympanocentesis skills by nurse practitioners in management of otitis media,” Journal of the American Academy of Nurse Practitioners, vol. 13, no. 11, pp. 524–529, 2001. 11930518 10.1111/j.1745-7599.2001.tb00019.x

[5] Hughes C. A., McMenamin P., Mehta V., Pillsbury H., and Kennedy D., “Otolaryngology workforce analysis,” The Laryngoscope, vol. 126, pp. S5–S11, 2016. 10.1002/lary.26238 27576957

[6] Freund T., Everett C., Griffiths P., Hudon C., Naccarella L., and Laurant M., “Skill mix, roles and remuneration in the primary care workforce: who are the healthcare professionals in the primary care teams across the world?,” International journal of nursing studies, vol. 52, no. 3, pp. 727–743, 2015. 25577306 10.1016/j.ijnurstu.2014.11.014

[7] Wu Z. , “Deep learning for classification of pediatric otitis media,” The Laryngoscope, vol. 131, no. 7, pp. E2344–E2351, 2021. 33369754 10.1002/lary.29302

[8] Elabbas A. I., Khan K. K., and Hortinela C. C., “Classification of Otitis Media Infections using Image Processing and Convolutional Neural Network,” in 2021 IEEE 13th International Conference on Humanoid, Nanotechnology, Information Technology, Communication and Control, Environment, and Management (HNICEM), 2021: IEEE, pp. 1–6.

[9] Choi Y., Chae J., Park K., Hur J., Kweon J., and Ahn J. H., “Automated multi-class classification for prediction of tympanic membrane changes with deep learning models,” Plos one, vol. 17, no. 10, p. e0275846, 2022. 36215265 10.1371/journal.pone.0275846 PMC9550050

[10] Habib A.-R. , “Evaluating the generalizability of deep learning image classification algorithms to detect middle ear disease using otoscopy,” Scientific reports, vol. 13, no. 1, p. 5368, 2023. 37005441 10.1038/s41598-023-31921-0 PMC10067817

[11] Alhudhaif A., Cömert Z., and Polat K., “Otitis media detection using tympanic membrane images with a novel multi-class machine learning algorithm,” PeerJ Computer Science, vol. 7, p. e405, 2021. 10.7717/peerj-cs.405 PMC7959604 33817048

[12] Akyol K., Uçar E., Atila Ü., and Uçar M., “An ensemble approach for classification of tympanic membrane conditions using soft voting classifier,” Multimedia Tools and Applications, vol. 83, no. 32, pp. 77809–77830, 2024.

[13] Kılıçarslan S., Diker A., Közkurt C., Dönmez E., Demir F. B., and Elen A., “Identification of multiclass tympanic membranes by using deep feature transfer learning and hyperparameter optimization,” Measurement, vol. 229, p. 114488, 2024.

[14] Camalan S. , “Digital Otoscopy With Computer-Aided Composite Image Generation: Impact on the Correct Diagnosis, Confidence, and Time,” Otolaryngology–Head and Neck Surgery. 10.1002/ohn.965 PMC11905175 39221462

[15] Camalan S. , “OtoPair: Combining right and left eardrum otoscopy images to improve the accuracy of automated image analysis,” Applied Sciences, vol. 11, no. 4, p. 1831, 2021.

[16] Camalan S. , “OtoMatch: Content-based eardrum image retrieval using deep learning,” Plos one, vol. 15, no. 5, p. e0232776, 2020. 32413096 10.1371/journal.pone.0232776 PMC7228122

[17] Binol H. , “SelectStitch: automated frame segmentation and stitching to create composite images from otoscope video clips,” Applied sciences, vol. 10, no. 17, p. 5894, 2020.

[18] Binol H. , “Digital otoscopy videos versus composite images: a reader study to compare the accuracy of ENT physicians,” The Laryngoscope, vol. 131, no. 5, pp. E1668–E1676, 2021. 33170529 10.1002/lary.29253 PMC8610175

[19] Lu H., Camalan S., Elmaraghy C., Moberly A. C., and Gurcan M. N., “A video classification method for diagnosing ear diseases using otoscope imaging,” in Medical Imaging 2025: Computer-Aided Diagnosis, 2025, vol. 13407: SPIE, pp. 758–766.

[20] Binol H., Niazi M. K. K., Elmaraghy C., Moberly A. C., and Gurcan M. N., “OtoXNet—Automated identification of eardrum diseases from otoscope videos: A deep learning study for video-representing images,” Neural Computing and Applications, vol. 34, no. 14, pp. 12197–12210, 2022.

[21] Shaikh N. , “Development and validation of an automated classifier to diagnose acute otitis media in children,” JAMA pediatrics, vol. 178, no. 4, pp. 401–407, 2024. 38436941 10.1001/jamapediatrics.2024.0011 PMC10985552

[22] Ebenezer J. P. , “HDR or SDR? A subjective and objective study of scaled and compressed videos,” IEEE Transactions on Image Processing, 2024. 10.1109/TIP.2024.3404890 38814774

[23] Lee D. Y. , “A subjective and objective study of space-time subsampled video quality,” IEEE Transactions on Image Processing, vol. 31, pp. 934–948, 2021. 10.1109/TIP.2021.3137658 34965209

[24] Choi L. K. and Bovik A. C., “Video quality assessment accounting for temporal visual masking of local flicker,” Signal Processing: image communication, vol. 67, pp. 182–198, 2018.

[25] Seshadrinathan K., Soundararajan R., Bovik A. C., and Cormack L. K., “Study of subjective and objective quality assessment of video,” IEEE transactions on Image Processing, vol. 19, no. 6, pp. 1427–1441, 2010. 20129861 10.1109/TIP.2010.2042111

[26] Sheikh H. R., Sabir M. F., and Bovik A. C., “A statistical evaluation of recent full reference image quality assessment algorithms,” IEEE Transactions on image processing, vol. 15, no. 11, pp. 3440–3451, 2006. 17076403 10.1109/tip.2006.881959

[27] Wang Z., Bovik A. C., Sheikh H. R., and Simoncelli E. P., “Image quality assessment: from error visibility to structural similarity,” IEEE transactions on image processing, vol. 13, no. 4, pp. 600–612, 2004. 15376593 10.1109/tip.2003.819861

[28] Wang Z., Simoncelli E. P., and Bovik A. C., “Multiscale structural similarity for image quality assessment,” in The Thrity-Seventh Asilomar Conference on Signals, Systems & Computers, 2003, 2003, vol. 2: Ieee, pp. 1398–1402.

[29] Sampat M. P., Wang Z., Gupta S., Bovik A. C., and Markey M. K., “Complex wavelet structural similarity: A new image similarity index,” IEEE transactions on image processing, vol. 18, no. 11, pp. 2385–2401, 2009. 19556195 10.1109/TIP.2009.2025923

[30] Wang Z. and Li Q., “Information content weighting for perceptual image quality assessment,” IEEE Transactions on image processing, vol. 20, no. 5, pp. 1185–1198, 2010. 21078577 10.1109/TIP.2010.2092435

[31] Zhang L., Zhang L., Mou X., and Zhang D., “FSIM: A feature similarity index for image quality assessment,” IEEE transactions on Image Processing, vol. 20, no. 8, pp. 2378–2386, 2011. 21292594 10.1109/TIP.2011.2109730

[32] Zhang F., Mackin A., and Bull D. R., “A frame rate dependent video quality metric based on temporal wavelet decomposition and spatiotemporal pooling,” in 2017 IEEE International Conference on Image Processing (ICIP), 2017: IEEE, pp. 300–304.

[33] Tu Z., Chen C.-J., Chen L.-H., Birkbeck N., Adsumilli B., and Bovik A. C., “A comparative evaluation of temporal pooling methods for blind video quality assessment,” in 2020 IEEE international conference on image processing (ICIP), 2020: IEEE, pp. 141–145.

[34] Ding K., Ma K., Wang S., and Simoncelli E. P., “Image quality assessment: Unifying structure and texture similarity,” IEEE transactions on pattern analysis and machine intelligence, vol. 44, no. 5, pp. 2567–2581, 2020. 10.1109/TPAMI.2020.3045810 33338012

[35] Ding K., Liu Y., Zou X., Wang S., and Ma K., “Locally adaptive structure and texture similarity for image quality assessment,” in Proceedings of the 29th ACM International Conference on multimedia, 2021, pp. 2483–2491.

[36] Sun W., Wang T., Min X., Yi F., and Zhai G., “Deep learning based full-reference and no-reference quality assessment models for compressed ugc videos,” in 2021 IEEE International Conference on Multimedia & Expo Workshops (ICMEW), 2021: IEEE, pp. 1–6.

[37] Lee J., Kim E., and Yoon S., “Anti-adversarially manipulated attributions for weakly and semi-supervised semantic segmentation,” in Proceedings of the IEEE/CVF conference on computer vision and pattern recognition, 2021, pp. 4071–4080.

